# The efficacy and safety of cadonilimab combined with lenvatinib for first-line treatment of advanced hepatocellular carcinoma (COMPASSION-08): a phase Ib/II single-arm clinical trial

**DOI:** 10.3389/fimmu.2023.1238667

**Published:** 2023-10-24

**Authors:** Qian Qiao, Chun Han, Sisi Ye, Juan Li, Guoliang Shao, Yuxian Bai, Aibing Xu, Meili Sun, Wei Wang, Jian Wu, Ming Huang, Lijie Song, Luke Huang, Ting Liu, Wei Liu, Zhongmin Maxwell Wang, Baiyong Li, Michelle Xia, Li Bai

**Affiliations:** ^1^ Chinese People’s Liberation Army (PLA) Medical School, Beijing, China; ^2^ Daytime Chemotherapy Center, Beijing Arion Cancer Center, Beijing, China; ^3^ Department of Medical Oncology, Chinese People’s Liberation Army (PLA) General Hospital, Beijing, China; ^4^ Intervention Department, Zhejiang Cancer Hospital, Hangzhou, China; ^5^ Department of Gastrointestinal Oncology, Harbin Medical University Cancer Hospital, Harbin, China; ^6^ Department of Medical Oncology, Nantong Tumor Hospital, Nantong, China; ^7^ Department of Medical Oncology, Central Hospital Affiliated to Shandong First Medical University, Jinan, China; ^8^ Department of Gastroenterology and Urology II, Hunan Cancer Hospital, Changsha, China; ^9^ Department of Hepatopancreatobiliary Surgery, The First Affiliated Hospital, Zhejiang University School of Medicine, Hangzhou, China; ^10^ Intervention Department, Yunnan Cancer Hospital&The Third Affiliated Hospital of Kunming Medical University&Yunnan Cancer Center, Kunming, China; ^11^ Department of Medical Oncology, The First Affiliated Hospital of Zhengzhou University, Zhengzhou, China; ^12^ Akeso Biopharma, Inc., Zhongshan, China

**Keywords:** cadonilimab, lenvatinib, BsA, bi-specific antibody, hepatocellular carcinoma, HCC

## Abstract

**Purpose:**

This multicenter, open-label, phase Ib/II study aimed to assess the efficacy and safety of cadonilimab, a humanized, tetravalent, bispecific antibody plus lenvatinib in first-line treatment of advanced hepatocellular carcinoma (aHCC).

**Methods:**

Patients with histologically confirmed aHCC were included to receive either 6 mg/kg cadonilimab every 2 weeks plus lenvatinib (cohort A) or 15 mg/kg cadonilimab every 3 weeks plus lenvatinib (cohort B). The primary endpoint was objective response rate (ORR) by RECIST v1.1, while the secondary endpoints were safety, progression-free survival (PFS), overall survival (OS), disease control rate (DCR), duration of response (DoR), and time to response (TTR).

**Results:**

A total of 59 patients were enrolled (31 in cohort A and 28 in cohort B). The median follow-up time was 27.4 months as of the data cutoff date (July 28, 2023). The ORR in cohort A was 35.5% (95% CI: 19.2, 54.6) and that in cohort B was 35.7% (95% CI: 18.6, 55.9), and the median DoR was 13.6 months (95% CI: 4.14, NE) and 13.67 months (95% CI: 3.52, NE), respectively. The median PFS was 8.6 months (95% CI: 5.2, 15.2) and 9.8 months (95% CI: 6.9, 15.2), respectively. The median OS was 27.1 months (95% C: 15.7, NE) for cohort A, while it was not reached for cohort B. Grade ≥ 3 treatment-related adverse events (TRAEs) were reported in 66.1% of patients, with serious TRAEs occurring in 39.0% of cases. Decreased platelet count (47.5%), proteinuria (45.8%), hypertension (44.1%), and white blood cell count (44.1%) were the most common TRAEs.

**Conclusion:**

This novel combination therapy showed promising efficacy and manageable toxicity that could provide an option in first-line setting of aHCC.

**Clinical Trial Registration:**

[www.ClinicalTrials.gov], NCT04444167.

## 1 Introduction

Hepatocellular carcinoma (HCC) is the sixth most prevalent cancer around the world and the fourth leading cause of cancer-related death (1). Approximately 72% of HCC patients are in Asia. China has the highest global incidence (2) with an estimate of more than half of the new cases and deaths that occurred (3). The vast majority of Chinese patients were infected with hepatitis B, which may related to poor prognosis (4, 5) with a median overall survival (OS) of 6–8 months and a 5-year survival probability of 10% to 18% (6, 7).

Immune checkpoint inhibitors (ICIs), targeted therapy, and their combinations are now being investigated as first-line therapies for advanced HCC (8–12). The combination of atezolizumab and bevacizumab demonstrated good antitumor activity in the phase III IMbrave150 investigation with an objective response rate (ORR) of 30% compared with that of 11% in patients treated with sorafenib (13). Multiple kinase inhibitors combined with immune checkpoint inhibitors also exhibited potential efficacy and tolerability for HCC patients (8, 9). The LEAP-002 study did not reach the primary goal, but it did show that the combination of lenvatinib and pembrolizumab resulted in the longest median OS of 21.1 months ever reported in first-line treatment of advanced HCC (8). The combination of camrelizumab and apatinib significantly improved progression-free survival (PFS) [HR = 0.52, 95% CI (0.41–0.65)] and OS [HR = 0.62, 95% CI (0.49–0.80)] compared to sorafenib (9). In a phase I research, a new combination regimen incorporating anti-CTLA-4 with anti-PD-L1 showed encouraging safety and initial efficacy (14). Based on the recent phase III HIMALAYA study, the FDA approved the combination of tremelimumab plus durvalumab for first-line treatment of unresectable HCC. This combination significantly improved the OS [HR = 0.78, 95% CI (0.65–0.92)] and has greater ORR than sorafenib (20.1% vs. 5.1%) with a median duration of response (DoR) of 22.3 months (10). Grade 3/4 treatment-related adverse events (TRAEs) occurred in 25.8% of cases, with serious adverse events (AEs) occurring in 17.5% of cases, which is consistent with the prior findings of dual anti-PD-1/CTLA-4 therapy (15), but with increased toxicity as compared to monotherapy (11).

Compared to monotherapy, combination therapy had a better prognosis and improved anti-tumor activity. Nonetheless, combination therapy tended to have higher incidence of adverse reactions, and this has been a major barrier to the widespread adoption of dual anti-PD-1/CTLA-4 therapy. Therefore, novel therapies are required to facilitate enhanced blockade of PD-1 and CTLA-4 with less toxicity in first-line treatment of HCC.

Cadonilimab is a first-in-class, humanized, bispecific antibody targeting PD-1 and CTLA-4 simultaneously with Fc null design (16). With such a molecular structure, cadonilimab could eliminate Fc-mediated antibody-dependent cellular cytotoxicity (ADCC), antibody-dependent cellular phagocytosis (ADCP), and antibody-dependent cytokine release (ADCR), all of which may increase the safety of this novel molecule in the treatment of cancer. Additionally, cadonilimab possesses 10-fold higher binding avidity with high density co-expression of the antigens PD-1 and CTLA-4 than with low-density expression of a single antigen. This could aid the retention of cadonilimab in areas with high antigen density such as tumor microenvironment compared to peripheral tissues. Cadonilimab was developed as a symmetric tetravalent bispecific antibody, constructed using Akeso Biopharma PD-1 antibody penpulimab (AK105) and Akeso Biopharma CTLA-4 antibody quavonlimab (AK107). It is composed of two IgG1 subclass heavy chains and two kappa subclass light chains covalently linked by disulfide bonds. Lenvatinib, a multiple receptor tyrosine kinase inhibitor, exhibits potent antiangiogenic properties by inhibiting vascular endothelial growth factor receptors (VEGFR), fibroblast growth factor receptors (FGFRs), RET, platelet-derived growth factor receptor-a (PDGFRa), and KIT. Based on the results of the phase III REFLECT research, lenvatinib was approved as first-line therapy for unresectable HCC (12). This phase Ib/II study was the first study to examine the novel cadonilimab plus lenvatinib combination regimen, which involved dual PD-1/CTLA-4 inhibitors and an antiangiogenic agent in treating advanced hepatocellular carcinoma (aHCC). It was predicted that the synergistic effect may lead to improved antitumor activity and a favorable safety profile.

## 2 Patients and methods

### 2.1 Study design and patients

This study was a multicenter, open-label, single-arm, phase Ib/II clinical trial of cadonilimab plus lenvatinib in patients with aHCC. This trial was conducted across nine centers in China. There are two cohorts in this study. Three patients in cohort A received cadonilimab (C) 6 mg/kg intravenously on day 1, every 2 weeks (Q2W) plus lenvatinib (L) 8 mg (if body weight <60 kg) or 12 mg (if body weight ≥60 kg) orally once daily; if no DLTs were reported within 28 days after first dose, then subjects were further enrolled to 30. Three patients in cohort B received cadonilimab 15 mg/kg intravenously on day 1, every 3 weeks (Q3W), along with lenvatinib 8 mg (if body weight <60 kg) or 12 mg (if body weight ≥60 kg) orally once daily. If no DLTs were reported within 21 days of first dose, then enrollment could be continued to 30. An additional three subjects were required if one(1/3) of the first three subjects in either group displayed DLT. If ≥2 patients experienced DLTs in a total of six subjects, the sponsor and investigator will discuss and consider whether to continue, modify, or discontinue the study. The dosage group was determined based on the phase Ia dose escalation study from Australia and phase I/II clinical trial in China.

Eligible patients (≥18 years and ≤75 years) had a histologically confirmed HCC or clinically unresectable confirmed HCC according to American Association for the Study of Liver Diseases (AASLD) criteria, were classified as stage B or C by Barcelona Clinic Liver Cancer (BCLC) criteria, or ineligible for surgery or locoregional therapy for patients with BCLC stage B or progressed after surgery or locoregional therapy and not receiving previous systemic therapy, had at least one measurable target lesion (per RECIST v1.1), and had a Child-Pugh Score class A and Eastern Cooperative Oncology Group performance status of 0–1.

The trial was conducted in accordance with the International Conference on Harmonization guidelines for Good Clinical Practice and the Declaration of Helsinki. The institutional review board or independent ethics committee at Chinese People’s Liberation Army (PLA) General Hospital and other eight centers approved this trial.

### 2.2 End points and assessments

Safety was first analyzed by assessing DLTs in phase Ib. In the phase II study, the primary endpoint was ORR according to RECIST v1.1 per investigator assessment. Tumor assessment was performed every 6 weeks ( ± 7 days) within week 54 and then every 12 weeks ( ± 7 days). Patients who discontinued treatment for any reason other than disease progression or death should take tumor assessment on schedule until initiation of new anticancer treatment, death, radiographic progressive disease, loss to follow-up, or withdrawal of informed consent. Objective response should be re-evaluated within 4 weeks after first recorded. The secondary endpoints included PFS, OS, disease control rate (DCR), DoR, and time to response (TTR) according to RECIST v1.1 per investigator assessment.

### 2.3 Safety

AEs were monitored and graded by investigators using the National Cancer Institute Common Terminology Criteria for Adverse Events (NCI CTCAE), v5.0. AEs were collected until 30 days after the last dose or the start of the new anticancer treatment; serious AEs and immune-related AEs (irAE) were followed up to 90 days after the last dose or the start of the new anticancer treatment.

### 2.4 Statistical analyses

We estimated that a sample size of 30 patients would ensure at least 80% power to detect the ORR increase from 15% of anti-PD-1 monotherapy to 35% of cadonilimab combined with lenvatinib when the one-side type I error was 0.025.

The full-analysis set (FAS) was defined as enrolled patients who received at least one dose of study drug and has measurable target lesion (per RECIST v1.1) at baseline. The safety set (SS) was defined as enrolled patients who received at least one dose of study drug and has data recorded after treatment.

ORR and DCR with corresponding two-sided 95% CIs were estimated using the Clopper-Pearson method. Kaplan–Meier methods were applied to estimate DoR, PFS, and OS. Statistical analyses were performed using SAS software, version 9.4.

## 3 Results

### 3.1 Patients

**Figure 1:**
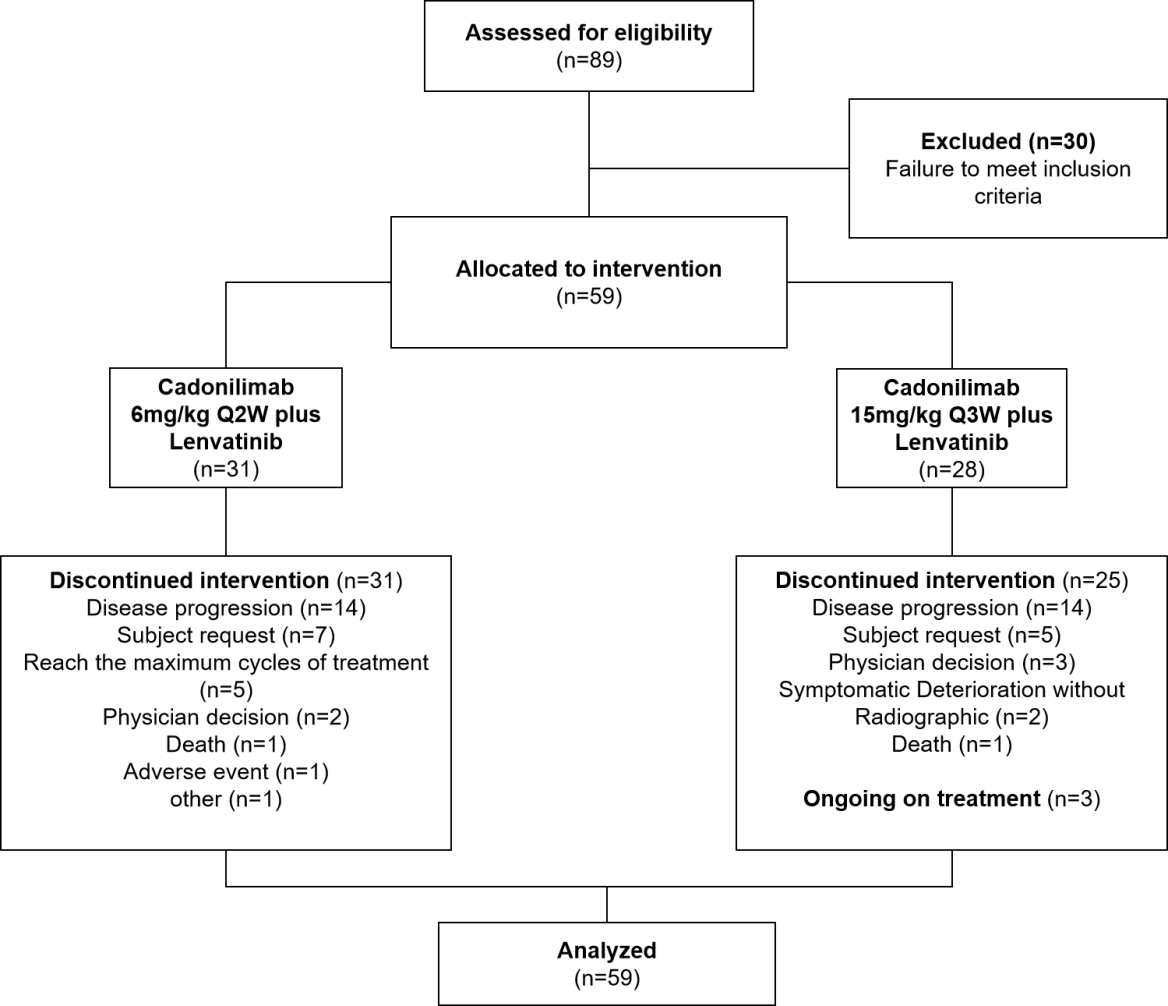
Diagram of patient disposition.

Between 7 July 2020 and 4 August 2021, 59 patients were enrolled (cohort A, *n* = 31; cohort B, *n* = 28). All enrolled patients were included in efficacy analyses and safety analyses (**Figure 1**). Three patients were still receiving treatment (cohort A, *n* = 0; cohort B, *n* = 3); 56 patients had discontinued treatment. The primary reason for discontinuation was disease progression for both groups (cohort A, *n* = 14; cohort B, *n* = 14). As of the date of data cutoff (28 July 2023), the median duration of follow-up was 27.4 months. Baseline demographics and clinical characteristics are summarized in **Table 1**.

**Table 1:** Baseline demographics and clinical characteristics.

Characteristics	Total *n* = 59	Cohort A *n* = 31	Cohort B *n* = 28
Age, years			
Median	56.3	53.4	56.6
Min–Max	24–72	32–72	24–72
Sex			
Male	48 (81.4)	27 (87.1)	21 (75)
Weight, kg			
<60	22 (37.3)	7 (22.6)	15 (53.6)
≥60	37 (62.7)	24 (77.4)	13 (46.4)
ECOG PS score			
0	31 (52.5)	22 (71)	9 (32.1)
1	28 (47.5)	9 (29)	19 (67.9)
Child–Pugh class			
A5	49 (83.1)	27 (87.1)	22 (78.6)
A6	10 (16.9)	4 (12.9)	6 (21.4)
HCC etiology			
HBV	54 (91.5)	30 (96.8)	24 (85.7)
HCV	0	0	0
Other	5 (8.5)	1 (3.2)	4 (14.3)
BCLC staging			
B	21 (35.6)	14 (45.2)	7 (25)
C	38 (64.4)	17 (54.8)	21 (75)
Extent of disease			
Macrovascular invasion	6 (10.2)	0	6 (21.4)
Extrahepatic metastases	32 (54.2)	17 (54.8)	15 (53.6)
AFP, μg/L			
<400	43 (72.9)	23 (74.2)	20 (71.4)
≥400	16 (27.1)	8 (25.8)	8 (28.6)
PD-L1 status			
TC/IC ≥ 1%	27 (45.8)	14 (45.2)	13 (46.4)
TC/IC < 1%	27 (45.8)	13 (41.9)	14 (50.0)
Unknown	2 (3.4)	2 (6.5)	0
Missing	3 (5.1)	2 (6.5)	1 (3.6)
Received locoregional therapy			
Yes	41 (69.5)	23 (74.2)	18 (64.3)
Percutaneous hepatic arterial chemoembolization	35 (59.3)	18 (58.1)	17 (60.7)
Ablation	13 (22)	11 (35.5)	2 (7.1)
Radiotherapy	1 (1.7)	1 (3.2)	0
Other	1 (1.7)	0	1 (3.6)
Received surgery			
Yes	33 (55.9)	19 (61.3)	14 (50)
No	26 (44.1)	12 (38.7)	14 (50)

Data are No. (%) unless otherwise indicated.

AFP, alpha-fetoprotein; BCLC, Barcelona Clinic Liver Cancer; ECOG, Eastern Cooperative Oncology Group; HBV, hepatitis B virus; HCC, hepatocellular carcinoma; HCV, hepatitis C virus; TC, tumor cell; IC, immune cell.

Median age was 56.3 (range: 24–72) years; 81.4% of subjects were men; 52.5% and 47.5% of subjects had ECOG PS of 0 and 1, respectively. Child-Pugh scores of A5 and A6 were reported for 83.1% and 16.9% of cases, respectively. Most of the HCC patients were HBV-related, accounting for 91.5% of subjects. BCLC stage B was noted in 35.6% of subjects and stage C was noted in 64.4%. There are 54.2% of patients who had extrahepatic metastases, and 10.2% of patients had macrovascular invasion; 27.1% of patients had an alpha fetoprotein level ≥400 μg/L.

### 3.2 Efficacy

**Figure 2:**
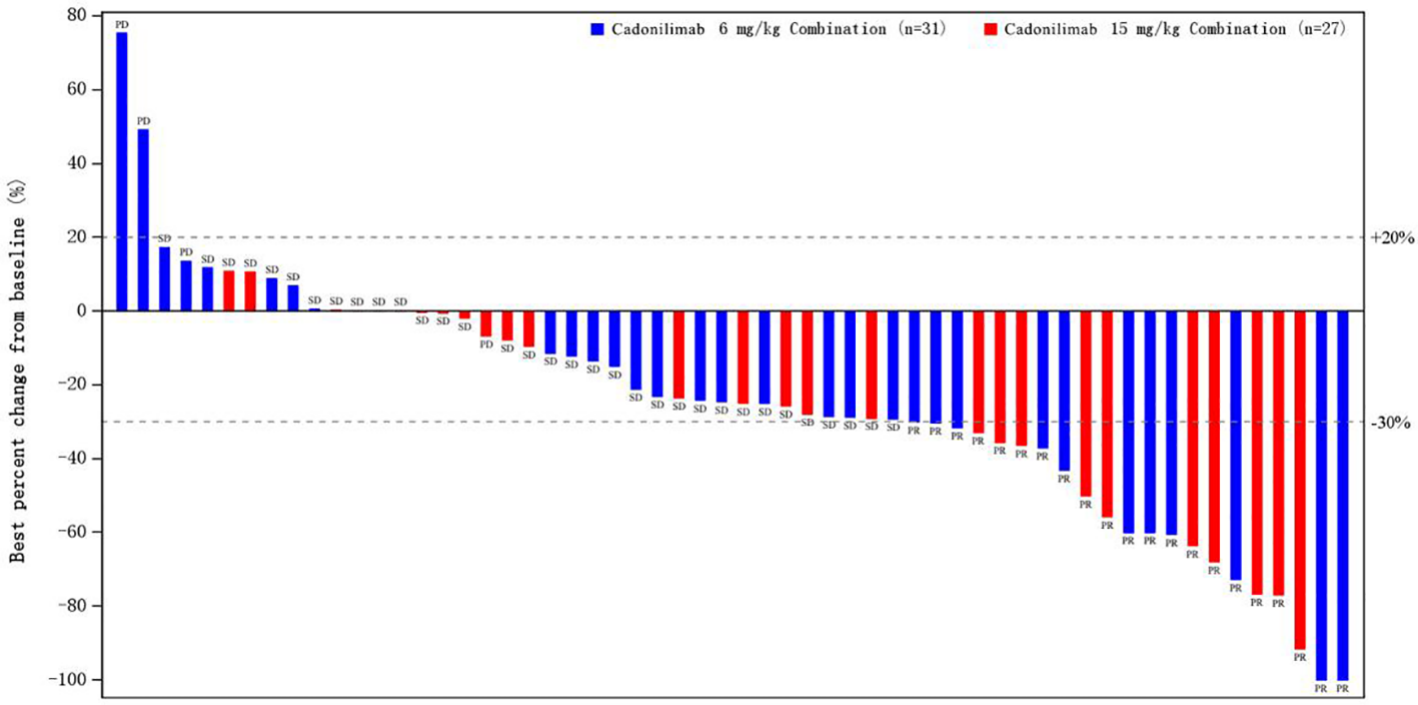
Percentage of best response for target lesion from baseline.

In cohort A and cohort B, ORR was 35.5% (95% CI: 19.2, 54.6) and 35.7% (95% CI; 18.6, 55.9), and median DoR was 13.6 months (95% CI: 4.14, NE) and 13.67 months (95% CI: 3.52, NE), respectively (**Table 2**). None of included patients achieved complete response (CR). Partial response (PR) was observed in 21 patients (35.6%), namely, 11 patients in cohort A and 10 patients in cohort B. DCR was reported in 90.3% (95% CI: 74.2, 98.0) of patients in cohort A and 92.9% (95% CI: 76.5, 99.1) of patients in cohort B. The median TTR was 2.76 months (range: 1.3–8.3) and 1.4 months (range:1.3–6.9) in cohort A and cohort B, respectively. Percentage of best response for target lesion from baseline is presented in **Figure 2**.

**Table 2:** Tumor response by investigator review per RECIST v1.1.

	Total *n* = 59 No. (%)	Cohort A *n* = 31 No. (%)	Cohort B *n* = 28 No. (%)
Best overall response			
Complete response	0 (0.0)	0 (0.0)	0 (0.0)
Partial response	21 (35.6)	11 (35.5)	10 (35.7)
Stable disease	33 (55.9)	17 (54.8)	16 (57.1)
Progressive disease	4 (6.8)	3 (9.7)	1 (3.6)
Not evaluable	0 (0.0)	0 (0.0)	0 (0.0)
Not applicable	1 (1.7)	0 (0.0)	1 (3.6)
Objective response	21 (35.6)	11 (35.5)	10 (35.7)
95% CI	23.6, 49.1	19.2, 54.6	18.6, 55.9
Disease control	54 (91.5)	28 (90.3)	26 (92.9)
95% CI	81.3, 97.2	74.2, 98.0	76.5, 99.1
Median time to response, months	1.61	2.76	1.4
Min-Max	1.3–8.3	1.3–8.3	1.3–6.9
Median duration of response, months	13.6	13.6	13.67
95% CI	5.55, NE	4.14, NE	3.52, NE

**Figure 3:**
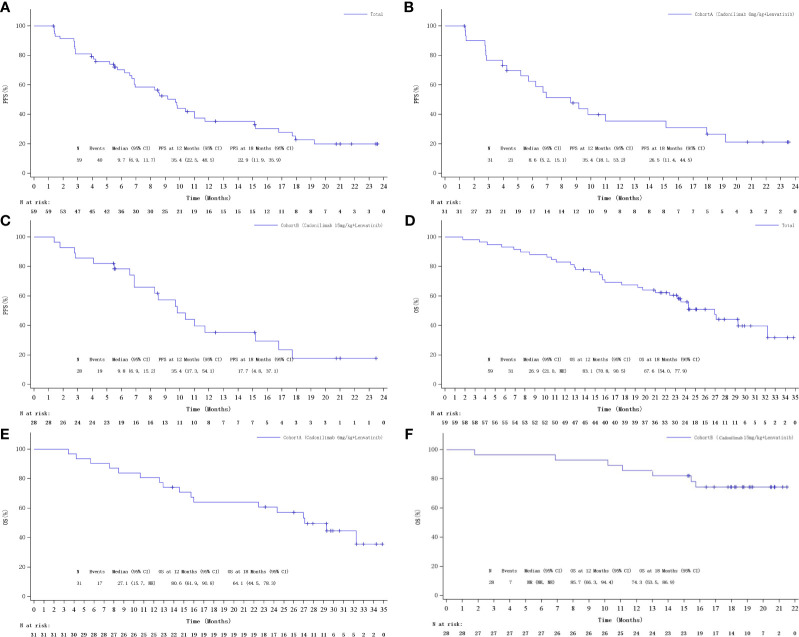
Kaplan–Meier curves of PFS: **(A)** Overall, **(B)** cohort A, and **(C)** cohort B; Kaplan–Meier curves of OS: **(D)** Overall, **(E)** cohort A, and **(F)** cohort B.

The median PFS was 9.7 months (95% CI: 6.9, 11.7 ) in total population (**Figures 3D**). The median PFS was 8.6 months (95% CI: 5.2, 15.2) and 9.8 months (95% CI: 6.9, 15.2) in cohorts A and B, respectively (**Figure 3C**). Kaplan–Meier estimated that 6-month PFS rate was 62.5% (95% CI: 42.5, 77.3) in cohort A and 78.4% (95% CI: 58.1, 89.7) in cohort B. The 12-month PFS rate was estimated to be observed in 35.4% (95% CI: 18.1, 53.2) and 35.4% (95% CI: 17.3, 54.1) of patients in cohorts A and B, respectively. The median OS was 26.9 months (95% CI: 21.0, NE) in total population (**Figures 3D**). The median OS was 27.1 months (95% CI: 15.7, NE) for cohort A, while it was not reached for cohort B (**Figures 3E, F**). Kaplan–Meier estimated that the 6-month OS rates were 90.3% (95% CI: 72.9%, 96.8%) and 96.4% (95% CI: 77.2%, 99.5%), the 12-month OS rates were 80.6% (95% CI: 61.9, 90.8) and 85.7% (95% CI: 66.3, 94.4), and the 18-month OS rates were 64.1% (95% CI: 44.5, 78.3) and 71.4% (95% CI: 50.9, 84.6), respectively, for cohort A and cohort B.

### 3.3 Safety

No DLTs were reported in the three patients enrolled in the DLT phase of each cohort. All patients experienced at least one TRAE (**Table 3**). The most common any grade TRAEs were decreased platelet count (47.5%), proteinuria (45.8%), hypertension (44.1%), decreased white cell count (44.1%), weight loss (40.7%), and decreased neutrophil count (40.7%). Grade ≥3 TRAEs occurred in 66.1% of patients with the most common grade ≥3 TRAEs being hypertension (20.3%), decreased platelet count (8.5%), increased alanine aminotransferase (6.8%), rash (6.8%), and decreased appetite (6.8%) (**Table 4**).

**Table 3:** Summary of TRAEs.

	Total (*n* = 59) No. (%)	Cohort A (*n* = 31) No. (%)	Cohort B (*n* = 28) No. (%)
Any	59 (100)	31 (100)	28 (100)
Grade ≥ 3	39 (66.1)	21 (67.7)	18 (64.3)
Serious TRAEs	23 (39.0)	10 (32.3)	13 (46.4)
Led to treatment discontinuation	7 (11.9)	4 (12.9)	3 (10.7)
Led to death	3 (5.1)	2 (6.5)	1 (3.6)

TRAE, treatment-related adverse event.

**Table 4:** TRAEs of any grade occurring in ≥20%.

	Total *n* = 59 No. (%)		Cohort A *n* = 31 No. (%)		Cohort B *n* = 28 No. (%)	
TRAEs	All Grades	Grade ≥3	All Grades	Grade ≥3	All Grades	Grade ≥3
Decreased platelet count	28 (47.5)	5 (8.5)	17 (54.8)	4 (12.9)	11 (39.3)	1 (3.6)
Proteinuria	27 (45.8)	2 (3.4)	10 (32.3)	1 (3.2)	17 (60.7)	1 (3.6)
Hypertension	26 (44.1)	12 (20.3)	15 (48.4)	8 (25.8)	11 (39.3)	4 (14.3)
White blood cell count decreased	26 (44.1)	2 (3.4)	17 (54.8)	1 (3.2)	9 (32.1)	1 (3.6)
Weight loss	24 (40.7)	1 (1.7)	15 (48.4)	1 (3.2)	9 (32.1)	0 (0.0)
Decreased neutrophil count	24 (40.7)	3 (5.1)	17 (54.8)	2 (6.5)	7 (25.0)	1 (3.6)
Alanine aminotransferase increased	22 (37.3)	4 (6.8)	15 (48.4)	3 (9.7)	7 (25.0)	1 (3.6)
Aspartate aminotransferase increased	22 (37.3)	2 (3.4)	15 (48.4)	1 (3.2)	7 (25.0)	1 (3.6)
Hypothyroidism	21 (35.6)	0 (0.0)	10 (32.3)	0 (0.0)	11 (39.3)	0 (0.0)
Blood bilirubin increased	19 (32.2)	1 (1.7)	10 (32.3)	1 (3.2)	9 (32.1)	0 (0.0)
Diarrhea	19 (32.2)	3 (5.1)	13 (41.9)	1 (3.2)	6 (21.4)	2 (7.1)
Anemia	17 (28.8)	0 (0.0)	12 (38.7)	0 (0.0)	5 (17.9)	0 (0.0)
Rash	15 (25.4)	4 (6.8)	12 (38.7)	3 (9.7)	3 (10.7)	1 (3.6)
Fatigue	14 (23.7)	0 (0.0)	11 (35.5)	0 (0.0)	3 (10.7)	0 (0.0)
Hypoalbuminemia	13 (22.0)	0 (0.0)	3 (9.7)	0 (0.0)	10 (35.7)	0 (0.0)
Blood thyroid stimulating hormone increased	12 (20.3)	0 (0.0)	6 (19.4)	0 (0.0)	6 (21.4)	0 (0.0)
Decreased appetite	12 (20.3)	4 (6.8)	8 (25.8)	2 (6.5)	4 (14.3)	2 (7.1)
Palmar metatarsal redness syndrome	12 (20.3)	0 (0.0)	6 (19.4)	0 (0.0)	6 (21.4)	0 (0.0)

Serious AEs (SAEs) and treatment-related SAEs were reported in 42.4% of patients and 39.0% of patients, respectively. The most common SAEs were disease progression (6.8%), liver injury (6.8%), and adrenal insufficiency (5.1%). TRAEs leading to treatment discontinuation were reported in 11.9% of patients. TRAEs that led to death occurred in two patients (6.5%) in cohort A and one patient (3.6%) in cohort B; all three patients (5.1%) died due to disease progression.

## 4 Discussion

To our knowledge, the present study first reported the efficacy and safety of bi-specific antibody (PD-1/CTLA-4) cadonilimab plus lenvatinib combination therapy in first-line treatment of advanced HCC. Our findings suggested that this combination regimen had promising efficacy and durable response. The toxicity was manageable and no unexpected safety signal was identified.

Cadonilimab plus lenvatinib demonstrated the encouraging efficacy in survival outcome analysis with a median PFS of 9.7 months and median OS was not reached. In comparison to prior findings, lenvatinib monotherapy demonstrated a median PFS of 7.4 months (12) and the phase III HIMALAYA study of the CTLA-4 inhibitor tremelimumab combined with the PD-L1 inhibitor durvalumab had a median PFS of 3.8 months (10). In terms of immune checkpoint inhibitor/antiangiogenic agent combinations, the phase III LEAP-002 study of lenvatinib plus pembrolizumab combination therapy exhibited a median PFS of 8.2 months (8), while a phase III study of camrelizumab plus apatinib showed a median PFS of 5.6 months (9). The combination of cadonilimab plus lenvatinib resulted in the longest PFS ever recorded in the first-line therapy of HCC and was associated with prolonged survival. Our results also suggested that the high dose of cadonilimab possibly translated into improved progression-free survival (mPFS: 8.6 months in cohort A vs. 9.8 months in cohort B).

Immunotherapy was found to be capable of providing durable and sustainable response, which resulted in substantial survival tails in the KM curves of OS. Despite the fact that the median OS was not reached in the present study, tumor response exhibited superior efficacy with an ORR of 35.6%, a median DoR of 13.6 months, and a median TTR of 1.61 months. The ORR was numerically superior to dual anti-PD-1/CTLA-4 therapy (10) and camrelizumab plus apatinib (9). The median DoR was consistent with dual anti-PD-1/VEGF therapy (8, 9). The median TTR was 2.76 months in cohort A whereas patients who received 15 mg/kg Q3W cadonilimab therapy achieved a median TTR of 1.4 months. The promising efficacy may be related to the combination of bi-specific antibodies (cadonilimab) and antiangiogenic agent (lenvatinib), which can inhibit PD-1, CTLA-4, VEGFR, FGFR, RET, PDGFRa, and KIT simultaneously. Meanwhile, anti-VEGF monoclonal antibodies can enhance T-cell-mediated tumor cell killing effect of immune checkpoint inhibitors such as anti-PD-1/CTLA-4 by promoting tumor vascular normalization, reversing VEGF-mediated tumor immunosuppression, and down-regulating MDSCs and Treg to regulate the tumor immune microenvironment. Comparison among different studies should be interpreted with caution; however, the present study suggested that cadonilimab plus lenvatinib might be an efficacious treatment option for first-line treatment of advanced HCC. These results suggested that HCC is sensitive to CTLA-4/PD-1 blockade, as previously suggested in nivolumab plus ipilimumab and tremelimumab plus durvalumab.

This study reported high rates of AEs with cadonilimab and lenvatinib combination therapy; however, the toxicity profile was consistent with the known safety profile of each drug and the underlying disease (12, 14). Cadonilimab-related AEs were tolerated when compared with other anti-CTLA-4/PD-1/PD-L1 combinations and consistent with published monotherapies (12, 14, 15). The incidence of grade ≥3 TRAEs and SAEs was similar to camrelizumab plus apatinib and pembrolizumab plus lenvatinib, which suggested that cadonilimab with bi-specific antibodies did not increase the toxicity in HCC patients (17, 18). The most frequent any-grade TRAEs were decreased platelet count, proteinuria, hypertension, decreased white cell count, and decreased neutrophil count. Specifically, decreased platelet count, decreased white cell count, and decreased neutrophil count are the most frequent TRAEs in cohort A, whereas proteinuria, hypertension, and hypothyroidism are the most frequent TRAEs that occurred in cohort B. However, in the 39 patients (66.1%) with grade ≥3 TRAE, only grade ≥3 hypertension occurred in >10% of patients, which was consistent with other antiangiogenic agent-containing regimens (9, 17). CheckMate-040 has demonstrated that the combination regimen of nivolumab (1 mg/kg) and high-dose ipilimumab (3 mg/kg) in advanced HCC resulted in the longest median OS (22.8 months) but were also associated with high rates of immune toxicity. In CheckMate-040, the discontinuation rate related to TRAEs was 22%, and among 10 patients who had a hepatic immune-mediated AE, 70% of cases received high-dose systemic corticosteroids (15). In the present study, approximately 11.9% of patients discontinued treatment owing to TRAEs, three patients (5.1%) died because of progressive disease, and the safety profile appeared favorable; only 32.2% of patients needed systemic steroids because of TRAEs. These rates were comparable to those reported in monotherapy studies of each agent in advanced HCC, suggesting that the toxicity profile of this regimen is manageable with appropriate monitoring, treatment interruption, or dose modifications. Cadonilimab’s higher binding avidity in a tumor-like setting and Fc-null design may result in better drug retention in tumors and contribute to improved safety while achieving anti-tumor activity.

A limitation of this study was the absence of a standard therapy as the control arm. As an open-label, phase Ib/II study, we enrolled a relatively small sample size that was not powered to detect differences between subgroups regarding the primary endpoint objective response.

## 5 Conclusion

In conclusion, cadonilimab combined with lenvatinib showed encouraging antitumor activity and manageable toxicity, which was a novel therapy and might provide an efficacious alternative option in first-line setting of advanced HCC.

## Data availability statement

The original contributions presented in the study are included in the article material. Further inquiries can be directed to the corresponding author.

## Ethics statement

The studies involving human participants were reviewed and approved by the ethics committee of the Chinese PLA General Hospital, The First Affiliated Hospital of Zhengzhou University, Harbin Medical University Cancer Hospital, Jinan Central Hospital, Zhejiang Cancer Hospital, The First Affiliated Hospital, Zhejiang University School of Medicine, Nantong Tumor Hospital, Hunan Cancer Hospital, and Yunnan Cancer Hospital. The studies were conducted in accordance with the local legislation and institutional requirements. The participants provided their written informed consent to participate in this study.

## Author contributions

LB, QQ, and CH conceived and designed the study. Equal contributions were made by QQ and CH. All authors recruited patients and collected data. All authors interpreted the data and reviewed and approved the final version of the submitted report and are accountable for all aspects of the report.
